# Transcranial Stimulation for the Treatment of Stimulant Use Disorder

**DOI:** 10.3390/neurolint15010021

**Published:** 2023-02-27

**Authors:** Amber N. Edinoff, Saveen Sall, T. Dean Roberts, Henry H. Tomlinson, Lenise G. Soileau, Eric D. Jackson, Kevin S. Murnane, Danielle M. Wenger, Elyse M. Cornett, Jaime Toms, Deepak Kumbhare, Adam M. Kaye, Alan D. Kaye

**Affiliations:** 1 Department of Psychiatry, Harvard Medical School, Massachusetts General Hospital, Boston, MA 02114, USA; 2 Louisiana Addiction Research Center, Shreveport, LA 71103, USA; 3 Department of Psychiatry and Behavioral Medicine, Louisiana State University Health Science Center at Shreveport, Shreveport, LA 71103, USA; 4 College of Pharmacy, University of Louisiana Monroe, Monroe, LA 71201, USA; 5 Department of Anesthesiology, Medical University of South Carolina, Charleston, SC 29425, USA; 6 School of Medicine, Louisiana State University Health Science Center at Shreveport, Shreveport, LA 71103, USA; 7 College of Medicine—Phoenix, University of Arizona, Phoenix, AZ 85004, USA; 8 Department of Pharmacology, Toxicology & Neuroscience, Louisiana State University Health Science Center at Shreveport, Shreveport, LA 71103, USA; 9 Department of Anesthesiology, Louisiana State University Health Science Center at Shreveport, Shreveport, LA 71103, USA; 10 Department of Neurosurgery, Louisiana State University Health Science Center at Shreveport, Shreveport, LA 71103, USA; 11 Department of Pharmacy Practice, Thomas J. Long School of Pharmacy and Health Sciences, University of the Pacific, Stockton, CA 95211, USA

**Keywords:** transcranial magnetic stimulation, neuromodulation, addiction, stimulants, cocaine, methamphetamine

## Abstract

The increasing prevalence of stimulant use disorder (StUD) involving methamphetamine and cocaine has been a growing healthcare concern in the United States. Cocaine usage is associated with atherosclerosis, systolic and diastolic dysfunction, and arrhythmias. Furthermore, approximately one of every four MIs is cocaine-induced among patients aged 18 to 45. Methamphetamine use has been associated with nerve terminal damage in the dopaminergic system resulting in impaired motor function, cognitive decline, and co-morbid psychiatric disorders. Current treatment options for StUD are extremely limited, and there are currently no FDA-approved pharmacotherapies. Behavioral interventions are considered first-line treatment; however, in a recent meta-analysis comparing behavioral treatment options for cocaine, contingency management programs provided the only significant reduction in use. Current evidence points to the potential of various neuromodulation techniques as the next best modality in treating StUD. The most promising evidence thus far has been transcranial magnetic stimulation which several studies have shown to reduce risk factors associated with relapse. Another more invasive neuromodulation technique being studied is deep-brain stimulation, which has shown promising results in its ability to modulate reward circuits to treat addiction. Results showing the impact of transcranial magnetic stimulation (TMS) in the treatment of StUD are limited by the lack of studies conducted and the limited understanding of the neurological involvement driving addiction-based diseases such as StUD. Future studies should seek to provide data on consumption-reducing effects rather than craving evaluations.

## 1. Introduction

The use of illicit stimulants such as methamphetamines and cocaine, and the misuse of prescription stimulants, have been a growing healthcare concern in the United States for many decades. Misuse of these substances can lead to development of a stimulant use disorder (StUD). A steady rise in availability and manufacturing of methamphetamines, along with increased cocaine use in adults and adolescent populations, continues to perpetuate this healthcare problem [1,2]. A study assessing the prevalence of stimulant use for medical purposes estimated a 79.8% increase in stimulant use from 2013 to 2018 [3].

While prescription stimulants have been proven to be an effective treatment option for Attention Deficit Hyperactive Disorder (ADHD), legitimate concerns have come with studies showing high levels of medical misuse, nonmedical use, and stimulant diversion [4]. The increased misuse of prescription stimulants was assessed in a short-term study which showed that the estimated number of emergency department visits involving ADHD prescription stimulants had steadily increased among those aged 18 years and older from 13,379 visits in 2005 to 31,244 visits in 2010 [5]. These epidemiological findings represent the growing threat of stimulant misuse, which demands action.

Behavioral therapy remains the only therapeutic option with proven efficacy for treating StUD. However, stimulants are often associated with high levels of relapse, and behavioral therapies are largely ineffective in the long term. While behavioral therapy can provide individuals with initial coping strategies, other treatment modalities should be utilized to prolong stimulant abstinence. Currently, evidence accessing various pharmacological treatments shows little to no significant efficacy in treating StUD. Neuromodulation is a new form of treatment that has been utilized in neurology and psychiatry and is continuously being studied for its efficacy as a future treatment for addiction-related disorders. One important neuromodulation technique known as transcranial magnetic stimulation (TMS) has provided evidence for decreased cravings among cocaine users. This paper aims to review the possibility of the use of TMS for the treatment of StUD.

### 1.1. Cocaine

Acute administration of cocaine has profound effects on neuronal activity and cerebral blood flow [6]. Cocaine binds to the monoamine transporters for dopamine, norepinephrine, and serotonin within the synaptic cleft. As a stimulant, these transporters’ binding inhibits the monoamines’ reuptake, which enhances post-synaptic activity [7]. The elevated level of dopamine then activates dopaminergic G-protein-coupled receptors (GPCRs) in the mesocorticolimbic dopamine pathway, which is responsible for the reinforcing properties of the drug [8]. The transcriptional regulator ∆FosB, which modulates AMPA glutamate receptor synthesis, is stimulated by D_1_-receptors. ∆FosB elevates the glutamate receptor subunit GluR1 in the ventral tegmental area following discontinuation of cocaine and could lead to addiction [9]. However, it is important to recognize that cocaine affects organ system beyond the brain, including the cardiovascular system, and can lead to changes in cerebral blood flow, vasoconstriction, vasculitis, atherosclerosis, systolic and diastolic dysfunction, and arrhythmias [10].

### 1.2. Methamphetamine

Methamphetamine exhibits profound addictive stimulant properties and strong dysregulation of neural circuitry [11]. It readily passes through the blood–brain barrier (BBB) to provoke rapid and intense euphoria [12]. As a sympathomimetic, methamphetamine increases heart rate, blood pressure, body temperature, heightens alertness, and decreases appetite. Administration of amphetamines stimulates the release of the monoamines norepinephrine, serotonin, and dopamine, greatly enhancing their synaptic levels [13]. Euphoric effects of methamphetamine are attributed to the overstimulation of dopamine. Chronic administration of methamphetamine contributes to nerve terminal damage in the dopaminergic system leading to potential motor deficits such as: impaired motor function, cognitive decline, anxiety, depression, hallucinations, violent behaviors, and delusions [14,15,16]. Users often experience immediate effects such as methamphetamine-induced restlessness, insomnia, and irritability. Continued use of methamphetamine can render the user with unwanted side effects such as dyskinesia and other movement side effects. The acute withdrawal phase begins with symptoms lasting between seven and ten days, peaking around 24 h after the last use. Withdrawal symptoms include dysphoria, fatigue, vivid and unpleasant dreams, increased appetite, and psychomotor alterations. The severity of symptoms is use-dependent and often varies with age and dependence [17]. As with cocaine, methamphetamine affects many organ systems beyond the brain, notably the cardiovascular system [18,19].

## 2. Current Treatment of StUD

Current public health concerns regarding the increased use of stimulants in the United States present ongoing efforts to unveil new protocols for diagnosing and treating StUD. Unlike opioid use disorders, current treatment options for StUD are very limited and only include behavioral interventions with no known FDA-approved pharmacotherapies. Over the years, suggested pharmaceutical treatments have been used off-label but have not provided any statistical effectiveness among various studies. A multi-center study assessed the use of bupropion and injectable naltrexone, but the authors could not reach a definite conclusion that this medication combination was superior to the placebo [20]. Nevertheless, advances in neurobiology and neurotechnology have allowed researchers to better grasp the neuropathological basis of dependence associated with stimulant use [21,22]. These advances have provided a greater opportunity for the future development of pharmacological interventions which could be used in conjunction with known effective behavioral therapies [23].

### 2.1. Current Behavioral Interventions for StUD

Behavioral therapies have been influential in treating many forms of drug dependence. In treating StUD, behavioral interventions remain the only modality that has shown proven benefits through clinical studies. Four of these interventions include contingency management (CM), cognitive-behavioral therapy (CBT), community reinforcement approaches (CRA), and motivational interviewing (MI).

The most effective behavioral intervention used to treat StUD currently is CM. This intervention has proven great efficacy in treating other substance use disorders, including alcohol, cannabis, nicotine, and opioids. During this intervention, material incentives are delivered to patients upon meeting expected goals for stimulant abstinence. This repeated reward stimulus trains the brain to reverse the desire for stimulant use. Individuals undergoing this treatment have displayed reduced psychological and physiological symptoms such as drug craving, mood/affect, and drug abstinence in patients diagnosed with StUD. In a systematic review and meta-analysis of 157 studies looking at treatment options for cocaine, CM programs, when compared to other psychotherapies, revealed provided the only significant likelihood that patients would provide a negative cocaine test [24]. Another study showed individuals receiving CM treatment compared to those who underwent psychosocial treatment for methamphetamine submitted more negative urine tests than those receiving standard care [25]. Additionally, this study provided evidence that CM procedures could be altered to allow for less frequent disbursements of reinforcements while still providing similar treatment efficacy. These studies are a few of many that point to CM as being a highly beneficial treatment option for StUD.

Another common behavioral treatment option for individuals diagnosed with StUD is CBT [26]. This approach seeks to reduce behaviors and thoughts associated with stimulant use through applying techniques such as motivational enhancement, relapse prevention, and stress management, which further help the patient cope with various drug-seeking triggers. The treatment formats for CBT often include individual therapy, group therapy, and, more recently, computer-based therapy. CBT has been extensively used for methamphetamine, though evidence of its effectiveness is still scarce. In a recent study, evidence for the use of CBT as a treatment for methamphetamine misuse was low and provided inconclusive findings as to the effectiveness or ineffectiveness of CBT [27]. The lack of evidence requires more research to address the effectiveness of CBT.

While some behavioral interventions have proven to be successful in treating individuals with StUD, it is necessary to find other treatment options that are more universally accessible and provide decreased relapse and better retention rates. Future studies should focus on the efficacy of using various behavioral treatment options in conjunction with other treatment modalities.

### 2.2. Pharmaceutical Interventions Being Studied

Much of the current research regarding StUD treatments involves potential pharmaceutical therapies targeting neurochemistry to reduce dependence and promote abstinence from stimulants. Some pharmacological agents being tested aim to reduce symptoms associated with StUD of a methamphetamine or amphetamine subtype. These medications include gabapentin, mirtazapine, bupropion, and rivastigmine, among others. Some of these drugs have effectively decreased psychiatric issues, which often provoke addictive behavior. However, these treatments have thus far failed to show consistent evidence of effectiveness between studies. Mirtazapine is currently FDA approved as an atypical antidepressant that is being studied as a potential treatment option for AMD by reducing stimulant craving. This drug option acts on the mesocorticolimbic system of the brain by inhibiting alpha-2-adrenergic receptors causing an increased release of norepinephrine, serotonin, and dopamine [28]. In a placebo-controlled experiment among cis-gender men and transgender women, mirtazapine treatment and counseling provided reductions in positive urine tests at 24 and 36 weeks of treatment [29]. Furthermore, comparing this study with others in a systematic review display similar findings with treatment groups showing small changes in methamphetamine use compared to placebo [14]. More robust studies looking at larger populations should be performed to render a conclusion about the effectiveness of mirtazapine [30]. Furthermore, pharmacotherapies for treating cocaine use disorders (CUD) show similar results with little effectiveness among many common drug classes [31]. Of the many studied pharmacotherapies, topiramate seems to be potentially effective in presenting abstinence. This could be achieved by the capacity of topiramate to facilitate GABAergic neurotransmission while inhibiting glutamatergic activity, which has been speculated to reduce the rewarding properties of cocaine and reduce craving [32]. Further studies should also evaluate these medications in conjunction with behavioral interventions.

## 3. Neuromodulation in Psychiatry

Medical technology advances have expanded knowledge and understanding of the neurophysiology and pathophysiology that drives many neurological and psychiatric diseases. Neuromodulation is a newer treatment option currently being utilized for many disorders. Some neuromodulation therapies are administered only using interventional techniques categorized as invasive or noninvasive. Some invasive treatment modalities are deep brain stimulation (DBS) and spinal cord stimulation (SCS). Non-invasive neuromodulation includes TMS. This review will focus on the use of TMS.

### 3.1. Transcranial Magnetic Stimulation

TMS was first introduced in 1985 as a noninvasive psychiatric diagnostic tool. Later, TMS exhibited its potential to target and treat many known psychiatric neuropathologies. Through a magnetic field, TMS produces an electric field that can penetrate the scalp and modulate cortical brain activity by targeting specific neuronal circuitry related to many disorders [33]. There are many different types and protocols for TMS such as continuous TMS, repetitive TMS (rTMS), and intermittent theta burst TMS just to name a few. In 2008, rTMS was the first to be approved and used for treating major depressive disorder (MDD) in individuals unresponsive to antidepressants. The approval of deep TMS (dTMS), which can penetrate deeper brain structures, allowed for the expansion of this modality for the treatment of obsessive-compulsive disorders (OCD), chronic migraine headaches, and most recently, the treatment of comorbid anxiety with MDD. These treatments’ future success and expansion depend on furthering the knowledge of neuronal circuits driving many psychiatric disorders. Furthermore, personalizing TMS to patient needs by introducing protocols with greater target specificity, varying frequencies, and treatment intensities can improve therapeutic efficacy [34].

### 3.2. Transcranial Magnetic Stimulation for Substance Use Disorders

With limited pharmacological treatments for StUD, the need for studies designed to assess the effectiveness of neuromodulation techniques, such as TMS, is important. The use of TMS to treat addiction-related disorders has shown promising results. One recent development has been the FDA clearance of dTMS therapy to treat short-term smoking cessation in adults. This marked a milestone for TMS therapy as it was the first time this modality was used to treat an addictive-related behavior. A double-blind, randomized control trial with 262 chronic smokers receiving active or sham TMS therapy five days a week for three weeks revealed that individuals in active and sham groups who underwent full treatment showed continuous quit rates of 28.4% and 11.7%, respectively [35]. Current and future studies are working to improve this treatment’s efficacy for smoking cessation and assess its effectiveness when performed in conjunction with other nicotine replacement therapies.

There have been studies to try to use neuroimaging to identify areas that are dysfunctional in those who use cocaine and use TMS to try to target these areas [36]. The idea is that these images could be used both diagnostically and prognostically in terms of treatment. The most used neuroimaging modality for this purpose is functional magnetic resonance imaging (fMRI). Though there are not many studies in the literature looking at using neuroimaging with TMS for cocaine use disorder, this is an exciting prospect because it can help pinpoint areas of dysfunction and increase the understanding of this substance use disorder [36].

It is important to note though that there are unknowns at this time regarding TMS and the treatment of substance use disorders [37]. These unknown fall into distinct categories which are cortical target selection, subcortical circuit engagement, optimizing rTMS sequences, rTMS as an adjuvant to existing treatments, manipulating brain states, and selecting outcome measures [37]. In terms of the unknown of cortical target selection, participant differences in anatomy and idiosyncrasies need to be accounted for and one target may not be the one size fits all option for all TMS treatments. More clinical decisions may need to be made to take these idiosyncrasies into account and more targets may need treatment. This leads to the second unknown which is subcortical circuit engagement. Once a cortical target is identified, then its subcortical circuit target needs to be validated. This would need to be done wither other measures such as EEG studies or fMRI studies. The amount of studies that look at this is very small so the generalizability is understanding to its full extent, which leads to this being known as one of the unknowns at this time. Another unknown is what is the optimized rTMS sequences because there could be individual differences in the effects of rTMS [37]. More research about how many pulses, for what amount of time, and for how many sessions needs to be done to make this unknown more known. The next unknown is the effect of rTMS as an adjuvant to existing interventions. This unknown would be easier to address in opioid use disorder since some patients are on methadone which requires daily clinic visits, however, there are no medications to treat cocaine use disorder so its combination with behavioral interventions should be studied. Another unknown is being able to manipulate the brain state. This physiological state should be taken into account; however, there are issues that may make this more complicated. One such issue is the use of multiple substances, which would make the manipulating of the brain state harder. The use of fMRI could help better understand the brain state in a multitude of substance use disorders. The last unknown is regarding selecting outcome measures [37]. Clinical interviews and craving scores are the usual measures of rTMS outcome measures. However, cravings may not be a good proxy for substance use as a symptom of disease because relapse may happen in the absence of cravings. Interviews may be hindered if rapport is not build between the research staff and the participant [37]. These unknowns are pointed out not necessarily as highlighted limitations but should be viewed as areas for improvement and further studies to better understand rTMS and its use in substance use disorders.

Animal and human studies have provided promising evidence that rTMS targeting the dorsolateral prefrontal cortex (DLPFC) may have the potential to alleviate craving in individuals with general substance dependence [38,39]. To bolster these findings, a randomized sham-control clinical trial using high-frequency rTMS to treat alcohol use disorders showed that stimulation of right DLPFC diminished both alcohol use and craving [40]. While these are promising findings, this evaluation requires further consideration as craving scales reflect a psychological construct which commonly leads to over-interpretation of results. Future studies should seek to provide data on consumption-reducing effect rather than craving evaluations.

## 4. Clinical Studies

### 4.1. Dorsal Lateral Prefrontal Cortex (DLPFC)

Research in the past decade shows promise for this technology to treat substance use disorders [41]. Different coils are used for TMS stimulation, and shapes include circular, butterfly, or figure-of-eight, elliptical, and a D-shape. Different shapes have different stimulation features [42]. In one study, 74 methamphetamine users were each randomly assigned to one of four different protocol groups. The first protocol used the excitatory stimulus mode (intermittent theta burst stimulation, iTBS) to stimulate the DLFPC; the second used inhibitory, continuous theta burst stimulation (cTBS) targeting the ventromedial prefrontal cortex (VMPFC); the third combined both the first and second settings; the fourth was a sham stimulation that used a coil where no magnetic field was generated. Eligibility criteria specified methamphetamine use within the last three months, males aged 18–49 who passed an in-person interview, and with no other serious neuropsychiatric diagnoses. Stimulation intensity was first set at 80% of the resting motor threshold (rMT) and gradually increased up to 100% rMT over the DLPFC or 110% rMT over the VMPFC. All subjects received ten sessions of treatment over two weeks, one session per day, five days per week. Researchers observed that all three TMS protocols significantly reduced craving for methamphetamine compared to the sham group. Patients undergoing the combined protocol (stimulation over the DLPFC and the VMPFC) showed a decreased time to treatment response compared to those in the protocol targeting only the DLPFC site. No serious adverse events were reported. The primary outcome was the change in craving scores, and the secondary outcome was efficacy. Treatment produced greater than or equal to 60% reduction in craving scores [42]. Limitations were identified. The study targeted the desired stimulation area by using EEG international 10–20 system positioning, which does not consider individual head shapes and can impact treatment efficacy. A common challenge in this study and others is that TMS causes a slight discomfort. However, sham stimulation does not. The small number of subjects limits the quality of results. Moreover, there can be variability in the same area of the brain in different illnesses, meaning that the frequency of the setting may need to be altered depending on what is being treated [42]. For example, depression studies show that high-frequency stimulation of the left DLPFC improves depression, but low-frequency stimulation of the left DLPFC improves sleep quality [43,44]. Overall, researchers felt this treatment regimen holds promise for the future treatment of addictive disorders, and further study is indicated [42].

Another common drawback is that most studies focus on males. One study, however, looked at 90 female methamphetamine users, ages 17–51, and compared rehab therapy with rehab plus TMS [45]. The study randomly assigned participants to a control group (32 patients) where they received routine addiction rehab therapy or an active treatment group (52 patients) that received the same standard rehab therapy plus 10 Hz TMS with 20 treatments over four weeks, administered five times per week [45]. Subjects were shown a video of methamphetamine use for five minutes, then rated a craving score on a scale of 0–100. Patients were evaluated the day before treatment (day 1), the first day after treatment completion (day 30), and a third time on day 60 or the 30th day after treatment completion. There was a 24-point difference in craving scores between the two groups on day 30 and a 21-point difference in craving scores on day 60. The effect of TMS treatment was found to be more pronounced among younger subjects with higher craving scores. Results showed that rTMS was effective in female methamphetamine users, and the effect lasted for at least 30 days after the 20-day treatment regimen [45].

In another study, rTMS was applied over the left DLPFC in 29 male long-term methamphetamine users, ages 21–55, with a history of weekly use over three years [46]. They were randomly assigned to either high-frequency rTMS (10 Hz) or sham rTMS over the left DLPFC for a two-choice oddball task. The sham condition was performed with a 1 Hz coil which was turned away from the skill at 90 degrees with only one edge resting on the scalp. This allows for the sensation of receiving rTMS since the participant feels the edge of the coil on their scalp; however, they do not receive any of the magnetic stimulation the rTMS group receives. The participant is usually not aware if they are receiving rTMS or not and this will control for any participant-based bias in results. Response times were noted to be significant based on methamphetamine users; however, it was noted that 10 Hz rTMS over the left DLPFC significantly decreased the slow response time and response errors by increasing the reaction time after errors were made. The 10 Hz rTMS intervention did not see the same increase in reaction times after correct responses. In the pretest, methamphetamine users who received the 10 Hz rTMS showed greater slowing on subsequent tasks following an error than healthy participants. Both groups were similar in the post-test. Researchers concluded that high-frequency rTMS at the left DLPFC could effectively adjust behavior in long-term methamphetamine users [46].

A randomized, single-blind pilot study with 83 methamphetamine-dependent males, age 18–60, abstinent in a long-term residential treatment program were randomly allocated by a computer-generated sequence into three groups: iTBS over the left DLPFC (active group), cTBS over the left DLPFC (active control group), or cTBS to the right DLPFC (active group). Treatment was administered to all patients twice daily over five days for ten sessions. Patients included in the study were required to have a primary StUD diagnosis of a methamphetamine subtype with a duration of at least one year and with using more than 0.1 g daily for a minimum of three months. A psychiatrist ruled out other severe psychiatric disorders. Exclusion criteria included history of other psychiatric disease, epilepsy, cardiac conditions, or contraindications to TMS, such as a metal implant in the skull. In the rehabilitation facility, all participants received standardized rehabilitation treatment, including daily physical exercise, supportive therapy for relapse prevention, and no medications. Nine patients did not complete the study, and no significant differences in demographics were found between those who did and did not complete it. TBS was applied using a coil, Figure 8. Intensity for each subject was calculated at 70% of the resting motor threshold. A single experimenter administered the intervention and was not blinded to group assignment. The participant and another experimenter who assessed the outcome were both blinded.

Researchers measured the primary outcome, cue–induced craving, and secondary outcomes of sleep, depression, anxiety, and impulsivity scores, as well as adverse effects. Seventy-four subjects completed five days of treatment. Adverse reactions included headache, neck pain, scalp tingling, itching, sleepiness, mood change, and difficulty concentrating. No severe adverse reactions were identified. Researchers identified a post-intervention beneficial effect on craving by iTBS of the left DLPFC and cTBS of the right DLPFC. Both treatments reduced craving. Constant TBS of the left DLPFC did not have the same effect. The secondary outcomes were not related to any improvement in methamphetamine craving. Sleep, depression, and anxiety all improved. However, measures for impulsivity were not impacted. Researchers concluded that there was variability in individual responses to left intermittent TBS treatment and that stimulating the left DLPFC or inhibiting the right DLPFC can reduce craving and drug consumption in these patients [38,47].

An August 2016 meta-analysis showed different left and right hemispheric roles for craving [48]. Researchers hypothesized that twice-daily TMS could be a feasible treatment for methamphetamine addiction [47]. The primary outcome measure was craving- determined by having patients watch video of methamphetamine use for five minutes, followed by a visual analog scale to evaluate craving scores ranging from 0 (no desire) to 100 (high desire) [47,49]. The same video was used before and after five days of treatment. Response to TMS was defined as a 50% reduction in symptoms defined as cravings. Researchers concluded that accelerated twice-daily TMS was feasible and tolerable to decrease craving and mood changes in methamphetamine-dependent patients who were abstinent [47]. The regimen also allowed them to reduce session length, meaning more patients could receive treatment. Twice daily left iTBS or right cTBS over five days reduced craving for methamphetamine. Researchers stressed the need for a larger study with a sham control to confirm findings. They also identified the need for a longer duration of clinical follow-up to determine duration of treatment efficacy and the relapse rate [47].

In another study of methamphetamine addicts, left or right-sided high and low-frequency rTMS effectively decreased cue-induced craving for methamphetamine [41]. Bilateral frontal hemispheres were effective in reducer craving when the patients received rTMS. This was thought to be due to an impact on the midbrain dopaminergic system creating alterations in prefrontal function or the restoration of plasticity of the brain. Fifty male methamphetamine users, ages 24 to 58, were randomly assigned to five groups—10 Hz left P3, 10 Hz left DLPFC, 10 Hz right DLPFC, 1 Hz left DLPFC, or 1 Hz right DLPFC. P3 is a mapped brain region that works with cognition-related brain information processing, intelligence and working memory and requires the interaction of large scale brain areas [50]. Subjects had been drug-free for two months, and there were no significant differences among the five groups in terms of cue induced cravings. Researchers found that high and low-frequency rTMS at either right or left DLPFC decreased craving scores immediately and after five days of continuous therapy.

In contrast, rTMS at P3 showed no similar effect. There were no significant differences in results of the four protocols. Researchers concluded that both high and low-frequency rTMS at the left or right DLPFC effectively managed cue-induced methamphetamine cravings [41].

### 4.2. Prefrontal Cortex (PFC)

Methamphetamine dependence is specifically associated with increased impulsivity attributed to several neural circuit changes [51]. The PFC plays a key role in execution and inhibition of behavior. It is involved in decision-making processes and the inhibition of risk-taking [51]. Patients who use methamphetamine show poor performance in working memory, cognitive control, attention, and decision-making processes, all closely associated with the PFC. Evidence has shown that noninvasive brain stimulation can reactivate the PFC and reduce cue-induced drug craving, drug use, and addiction to substances such as methamphetamine and cocaine [49,51,52,53]. A randomized, double-blinded clinical trial of 73 male methamphetamine users and 33 healthy male controls designed to identify if low-frequency rTMS over the left PFC could reduce impulsivity in methamphetamine patients assigned a two-choice oddball task [51]. Intervention effects were evaluated after a single session on day 1, 24 h later, after ten repeated sessions on day 11, and at a three-week follow-up on day 31. There were 36 patients in the sham group and 37 in the treatment group. They received daily TMS treatments for ten consecutive days. The primary outcome was impulse inhibition measured by accuracy on a two-choice task. Methamphetamine users showed greater impulsivity than the control group on the two-choice task, which impacted accuracy. A single session of 1 Hz rTMS over the left PFC increased the accuracy from 91.4% to 95.7%, and reaction time delay went from 50 ms to 77 ms. This was seen consistently after ten sessions of 1 Hz rTMS treatment. The behavioral improvement was maintained for at least three weeks after treatment and was coupled with a reduction in addictive symptoms measured by craving induced by drug cues. Researchers concluded that low-frequency rTMS at the left PFC can impact impulsivity in patients addicted to methamphetamine and can be part of a successful rehab program for methamphetamine addicts [51]. Though not understood fully in this context, it may be that the stimulation to the PFC may have allowed for neuronal rewiring to allow for better control of behaviors and, therefore, lead to a decrease in impulsive behaviors.

### 4.3. Help with Other Symptoms of Note

Drug abstinence is known to be associated with impaired sleep quality and mood changes in users. A double-blinded study with 105 male inpatients dependent on either heroin or methamphetamine with an average abstinence time of 6 months looked at the effect of rTMS on sleep quality and mood status during abstinence [54]. Patients were randomly divided into 10 Hz intervention (40 subjects), sham stimulation (40 subjects), and a control group that received no treatment (25 subjects). Five sessions of rTMS were given for six consecutive weeks for 180,000 pulses. Patients were assessed using the Pittsburgh sleep quality index, a self-rating scale for anxiety and depression, before and after six weeks of intervention. The study showed that six weeks of active rTMS treatment, compared to the sham and control groups, significantly improved sleep quality, depression, and anxiety in drug-dependent inpatients when used during abstinence and may have a beneficial effect on patients in treatment programs [54].

### 4.4. Cocaine and Neuromodulation

Chronic cocaine users have disruption in motor control [55]. During a simple task, cocaine users activate a larger area of the cortex than controls but have lower functional connectivity between the cortex and the dorsal striate, correlating with poor performance. Eighty-seven subjects (50 cocaine-dependent patients and 37 controls) were included in a study. fMRI information was gathered from a subset of 28 subjects who performed a finger-tapping task. TMS showed cocaine users had higher resting motor thresholds and higher intracortical facilitation than controls. There was no difference between the groups in the measure of cortical inhibition. The study aimed to determine motor cortex excitability in current cocaine users by applying a battery of single and paired-pulse TMS metrics with age-matched controls. A secondary goal was to determine if elevated BOLD signal in the left or right motor cortex of current cocaine users could be reproduced and if the observed aberrations in TMS parameters were associated with the BOLD signal in the motor cortex. The potential contribution of cortical atrophy with a higher brain-to-scalp distance was also evaluated. Researchers concluded that cocaine users had disrupted cortical facilitation measured by TMS and was related to an elevated BOLD signal. Cortical inhibition, however, was mostly intact [55].

TMS stimulates cortical and subcortical brain regions but to be effective, intact mono-synaptic connections are required [56]. Forty-nine cocaine users received single pulses of TMS to the frontal pole while BOLD data was collected. This technique is known as interleaved TMS/fMRI. Frontal pole TMS evoked activity in the striatum and the salience circuitry. This was the first study that demonstrated that the effect of TMS on subcortical activity is dependent on the structural integrity of the brain, suggesting that neuroimaging data can give biomarkers for TMS-induced stratum mobilization [56]. TMS changes activity at the cortical site and subcortical area targeted by the coil. DLPFC TMS can cause an increase or decrease in caudate dopamine [57].

The goal of this study was to understand the relationship between gray matter volume and white matter integrity with the use of TMS [57]. Fifty-one nontreatment seeking cocaine-dependent individuals, 26 male and 25 female with a mean age of 38, were recruited from the community and participated in one of the three multimodal imaging studies. Subjects were recruited through treatment programs and had a history of either cocaine or crack cocaine use by any route in the last five years. Exclusion criteria were the current use of prescription or illicit psychoactive drugs other than cocaine or marijuana or if the subject reported more than 100 lifetime uses of any drug of misuse other than cocaine. Subjects were excluded if they had any lifetime history of head trauma associated with loss of consciousness, pregnancy or breast-feeding, unstable medical illness, or a DSM-IV psychiatric disorder Axis I, or a current breath alcohol concentration greater than 0.002. Subjects in the study came to the imaging center and a resting motor threshold was determined for each individual. This is the minimum stimulation intensity applied over the hand area of the motor cortex that induces contraction of the contralateral abductor pollicis brevis muscle at least 50% of the time. The study showed that the effects of TMS are dependent on structural architecture of white and gray matter in the brain. Cocaine users have significantly lower white matter integrity in the frontal striatal and thalamic regions. The loss of white matter can impact TMS treatment in this population. Researchers found that white matter integrity between the side of the TMS stimulation and the subcortical target was critical to generate the required subcortical activity. This suggested that in patient populations with white matter diseases such as alcoholism, drug misuse, multiple sclerosis, or dementia, the effect of TMS on the striata would be greatest in those with higher levels of white matter integrity. Gray matter volume at the stimulation site was critical to activate the salience network. VMPFC gray matter volume reduction has been shown in cocaine users [57]. Prior studies have shown that gray matter volume can recover following sustained abstinence [58]. Researchers concluded that the efficacy of TMS could be greater after a period of abstinence [57]. Limitations of the study noted include 43 of the 49 participants being nicotine users and 35 being marijuana users. This precluded an absolute determination that the findings were specific only to cocaine use. They concluded that the results suggested white matter integrity and gray matter volume should be considered as the sources of variability when developing TMS as a treatment for populations with known changes in neural structure [56].

Researchers evaluated cocaine users to determine the daily tolerability and safety of three iTBS sessions [59]. Nineteen individuals with a use disorder related to cocaine received three open-label iTBS sessions daily. Of the 19 subjects recruited, 14 started the study, 2 did not tolerate TBS, and 3 were lost to contact. A total of 10 of the 14 participants received at least 26 of the 30 iTBS sessions and were considered completers of the study. Subjects received 60-min intervals between sessions for ten days over two weeks with a total of 30 iTBS sessions over the left DLPFC. Each session consisted of 600 pulses and 50 Hz bursts of three pulses. Cocaine use was evaluated before, during, and after the intervention, and again at one and four-week follow-ups.

There was no difference in cocaine use at the beginning of the study between those who did and did not complete the study. The first follow-up at week one showed a 54% reduction in cocaine use, and the second follow-up showed a 70% reduction in cocaine use. Interestingly, other drug use also decreased. Researchers noted that participants reported a reduced drive for cocaine use and an ability to stop using cocaine after initiating use which signified a reduction or change in compulsive drug use behavior that had been experienced before treatment. Subjects also noted they could not get as high as they had before treatment and felt this helped decrease their cocaine usage [59]. They noted anecdotal improvement in mood, and participants reported a reduction in craving. Researchers concluded that stimulation of the left DLPFC is a potential treatment intervention for cocaine addiction. The study was safe and overall well-tolerated. Researchers concluded that individuals with an active use disorder related to cocaine can tolerate accelerated iTBS during an intense two-week 30-session schedule. They also noted that iTBS did not have a high rate of negative side effects; and lastly, due to the small study size and the fact that it was open-label, no definitive conclusions could be made [59]. The researchers felt they laid the groundwork for larger studies to develop a treatment for StUD of a cocaine subtype.

Study to Rotolo et al. during a period of 2013–2020 looked at hair samples to monitor relapse in patients with cocaine use disorder during various stages of treatment with rTMS [60]. Patients were admitted to a specialized addiction treatment center in Padua, Italy and voluntarily underwent a protocol treatment with rTMS targeting the left DLPFC. During the clinical management, cocaine was assessed by urine screening, self-reports, and reports received by collateral information. rTMS was given twice daily for the first five days of treatment and then it was administered at weekly intervals in twice-daily sessions for 11 weeks. Hair samples of 6 cm in length were collected from nine patients and were analyzed in two different segments (proximal and distal) which represents two different hair growth stages and samples were only collected from the participants’ head. Hair samples demonstrated a reduction of cocaine use from the different of treatment to the end of treatment in most participants [60]. One case was not able to be fully analyzed since they self-terminated treatment early. This study focused on the use of hair samples to monitor treatment in patients undergoing treatment for cocaine use disorder, which they showed was a viable monitoring option; however, it also highlighted the rTMS could have been responsible for the reduction in cocaine use seen in these samples [60].

A retrospective observational study by chart review involved 284 outpatients, aged 18–70, who met DSM 5 criteria for a cocaine StUD—264 were male, and 20 were female. All patients underwent three months of rTMS. For the first five days, subjects received two sessions per day, after which they received treatment administered weekly, twice per day on each session day for 11 consecutive weeks. They received a 15 Hz frequency with 60 impulses per stimulation group, a 15-s break, and 40 total trains with a session duration of 13 min. TMS was administered weekly then monthly throughout the follow-up period. Patients received up to nearly three years of follow-up with a median follow-up of 164 days, and they were monitored for recurrent drug use. Results show that the median time to first relapse since beginning treatment was 91 days. Of the 284 patients, 147 maintained regular follow-ups through visits and phone calls allowing researchers to track patterns of cocaine use and abstinence, and 44% of patients were still abstinent at the end of monitoring. The clinic lost contact with approximately 50% of the monitored cases. The decreased frequency of rTMS sessions was not associated with increased lapses of cocaine use. The mean frequency of cocaine use in these patients in treatment was less than one day per month with the median days of cocaine use being zero. Researchers concluded that this is the first follow-up study to show that rTMS is accompanied by a long-lasting reduction in cocaine use in a large cohort of subjects. Adverse events were infrequent [61].

## 5. Conclusions

Given the greater accessibility of stimulants in many populations, it is vital to ensure that effective treatment options and diagnostic strategies are in place for StUD. Diagnostic strategies have improved with the advent of the DSM 5, but treatment options remain limited. Currently, studies seeking to expand treatment options for StUD are assessing three treatment modalities: pharmacological, behavioral, and neuromodulation, which is likely less known. While StUD symptoms in some individuals could be minimized by utilizing common psychiatric medications, there are currently no known pharmacotherapies providing consistent efficacy in treating StUD. Behavioral interventions are among the only effective modality used to treat StUD, and contingency management is currently the strongest evidence-based approach for treating StUD [62]. While behavioral therapies are effective in some individuals, the high rate of relapse contributes to the demand for other treatment modalities that can be used simultaneously.

Neuromodulation techniques have become increasingly popular in treating both neurological and psychiatric disorders. These modalities can potentially treat psychopathologies in a site-specific manner reducing side effects often associated with pharmacological agents and providing a more precision-based treatment approach. Currently, evidence points to the potential of various neuromodulation techniques as an effective modality in treating substance misuse. The most promising evidence thus far has been TMS as several studies have shown to reduce the risk factors associated with relapse. Results showing the impact that neuromodulation has on the treatment of StUD is limited by the lack of studies conducted and the understanding of the neurological involvement driving addiction-based diseases such as StUD. Another limitation is that most studies do not include women or include a very small number of women. Further studies with a more generalizable study population need to be performed with sham conditions to further evaluate neuromodulation’s effectiveness in these disorders.

## Data Availability

All data can be found in published studies on multiple academic indexing databases.
